# Associations between handgrip strength and hypertension in relation to circulating CD34-positive cell levels among Japanese older men: a cross-sectional study

**DOI:** 10.1186/s12199-021-00982-w

**Published:** 2021-06-04

**Authors:** Yuji Shimizu, Shin-Ya Kawashiri, Kenichi Nobusue, Hirotomo Yamanashi, Yasuhiro Nagata, Takahiro Maeda

**Affiliations:** 1 Department of Community Medicine, Nagasaki University Graduate School of Biomedical Sciences, Nagasaki-shi, Sakamoto 1-12-4, Nagasaki, 852-8523 Japan; 2 Department of Cardiovascular Disease Prevention, Osaka Center for Cancer and Cardiovascular Diseases Prevention, Osaka, Japan; 3 Department of Island and Community Medicine, Nagasaki University Graduate School of Biomedical Sciences, Nagasaki, Japan; 4 Department of General Medicine, Nagasaki University Graduate School of Biomedical Sciences, Nagasaki, Japan

## Abstract

**Background:**

A positive association between handgrip strength and blood pressure has been reported. Since these factors are linked to the condition of the endothelium, the activity of endothelial repair might influence the association between handgrip strength and hypertension.

**Methods:**

A cross-sectional study was conducted with 257 Japanese men aged 60–69 years who underwent an annual health checkup. As individuals with high level of circulating CD34-positive cells might show active endothelial repair, which plays an important role in vascular homeostasis, participants were stratified by circulating CD34-positive cell levels, using the median value of this population (0.96 cells/μL) as the cutoff.

**Results:**

Independent of known cardiovascular risk factors, for participants with a high CD34-positive cell, handgrip strength is significantly positively associated with hypertension (odds ratio and 95% confidence interval of hypertension for 1 standard deviation increment of handgrip strength were 1.85 (1.19, 2.88) but not for participants with a low CD34-positive cell (0.91 (0.61, 1.37)).

**Conclusion:**

The positive association between handgrip strength and hypertension is limited to high CD34-positive cells. This result may help clarify the role of vascular homeostasis in maintaining muscle strength.

## Introduction

Although previous studies have revealed a positive association between handgrip strength and blood pressure among older participants [1, 2], handgrip strength has been inversely associated with cardiovascular disease [3], whereas hypertension is a well-known cardiovascular risk factor [4]. These studies found an ambivalent association of handgrip strength with hypertension and cardiovascular disease.

Hypertension is known to be strongly associated with atherosclerosis [5, 6]. Previously, we reported that active endothelial repair, which results in atherosclerosis, might have a beneficial influence on maintaining muscle strength among hypertensive older Japanese [7, 8]. Therefore, even though hypertension is a well-known risk factor for cardiovascular disease [4], hypertensive older Japanese with active endothelial repair could show a beneficial effect on the maintenance of muscle strength, resulting in a positive association between handgrip strength and blood pressure.

Hematopoietic stem cells, such as CD34-positive cells derived from bone marrow, play a major role in vascular homeostasis [9–11], and a high circulating CD34-positive cell level categorized by the median value could indicate active endothelial repair [12–14].

Therefore, we hypothesized that the positive association between handgrip strength and hypertension is observed only in participants with high CD34-positive cells.

To clarify these associations, we conducted a cross-sectional study of 257 older Japanese men aged 60–69 who participated in an annual health checkup in 2014–2015.

## Methods

### Study population

This study was approved by the Ethics Committee of Nagasaki University Graduate School of Biomedical Sciences (project registration number: 14051404-11, approved October 31, 2019). All procedures performed in studies involving human participants were in accordance with the ethical standards of the institution research committee and the 1964 Helsinki Declaration and its later amendments for comparable ethical standards. Consent forms written in Japanese were made available to ensure a comprehensive understanding of the study objectives and were provided by all participants.

The study population comprised 274 Japanese participants aged 60–69 years from Goto City in the western part of Japan who attended an annual health checkup in 2014–2015. This annual checkup program was conducted by the local government and directed by the Ministry of Health, Labour, and Welfare in Japan. Based on this annual health check-up, we performed a CD34-positive cell-related survey to clarify the mechanism of aging, including the decrease in handgrip strength. The details of this survey have been described elsewhere [15].

Participants with a history of stroke (*n* = 14) and those without handgrip data (*n* = 3) were excluded from the study population.

The remaining participants, 257 men with a mean age of 65.4 (standard deviation (SD): 2.6), were enrolled in the study.

### Data collection and laboratory measurements

Information on clinical history such as history of stroke and taking anti-hypertensive medication and habitual status were obtained by trained interviewers.

Systolic and diastolic blood pressures on the right arm were recorded by trained examiners using a blood pressure measuring device (HEM-907; Omron, Kyoto, Japan) after at least 5 min of rest in a sitting position.

Hypertension was defined as systolic blood pressure ≥ 140 mmHg and/or diastolic blood pressure ≥ 90 mmHg [16]. Systolic hypertension was defined as systolic blood pressure ≥ 140 mmHg and diastolic hypertension as diastolic blood pressure ≥ 90 mmHg.

Body weight and height in bare feet and light clothing were measured using an automatic body composition analyzer (BF-220; Tanita, Tokyo, Japan). Body mass index (BMI) was calculated as (weight, kg)/(height, m)^2^.

Fasting blood samples were collected in a heparin sodium tube, an EDTA-2K tube, and a siliconized tube. White blood cell count (WBC), triglycerides (TG), HDL-cholesterol (HDLc), hemoglobin A1c (HbA_1C_), γ-glutamyltranspeptidase (γ-GTP), and creatinine were measured using standard laboratory procedures at SRL, Inc. (Tokyo, Japan). The glomerular filtration rate (GFR) was estimated according to an established method proposed by a working group of the Japanese Chronic Kidney Disease Initiative [17]. According to this adaptation, GFR (mL·min/1.73 m^2^) = 194 × (serum creatinine (enzyme method)) ^−1.094^ × (age) ^−0.287^.

Fresh samples from the heparin sodium tube were used within 24 h of collection to determine the number of circulating CD34-positive cells using BD Trucount^TM^ technology (Beckton Dickinson Biosciences; San Jose, CA), an accurate and reproducible single platform assay conforming to the International Society of Hematotherapy and Graft Engineering (ISHAGE) guidelines [18] and supported by automated software on the BD FACSCanto^TM^ II system. To measure CD34-positive cells, fresh samples (analyzed within 24 h of blood collection) were required. Therefore, the maximum number of samples measured in a day was limited to 20 samples and each sample took approximately 30 min to measure. Then, we limited the measurement of CD34-positive cells to men aged 60–69 years [15].

Handgrip strength was determined using a handgrip dynamometer (Smedley, Matsumiya Ika Seiki Seisakujo, Tokyo, Japan) as the grip strength from two measurements obtained for each hand, of which the maximum value was used.

### Statistical analysis

Since circulating CD34-positive cell levels divided by the median value of the study population determines the association between cardiovascular risk factors and hypertension [19, 20], study participants were stratified by circulating CD34-positive cell counts (median values) as described previously [5, 12–14, 19, 20].

Participants were classified into two groups based on the median value of circulating CD34-positive cells, with a cutoff value of 0.96 cells/μL.

To validate the study population in the present study, the Hosmer-Lemeshow test for goodness of fit was calculated.

Characteristics of the study population in terms of circulating CD34-positive cell levels were expressed as mean ± standard deviation for continuous values and proportion for medication status. The differences in the mean values or proportions of circulating CD34-positive cell levels were analyzed. Significant differences were evaluated with analysis of variance (ANOVA) for continuous variables and with the chi-squared test for proportion data.

Simple correlation analyses were performed to evaluate the correlation between the CD34-positive cell count and WBC count.

Logistic regression models were used to calculate odds ratios (ORs) and 95% confidence intervals (CIs) to investigate the association between handgrip strength and hypertension. In addition to contentious values of handgrip strength, tertile values of handgrip strength were also used to clarify the linear association between handgrip strength and hypertension. Adjustments for confounding factors were made using two models. In the first model (age-adjusted model), adjustments were made only for age. The second model (the multivariable model) included other, possibly confounding factors such as BMI (kg/m^2^), antihypertensive medication use (yes, no), HDLc (mg/dL), TG (mg/dL), γ-GTP (U/L),WBC (cells/μL), HbA1C (%), and GFR (mL/min/1.73 m^2^).

Although habitual status and medication status could influence endothelial activity, they are secondary to intervenous environments such as oxidative stress and inflammation. We did not consider these statuses as confounding factors, as in our previous study [13].

Since hypertension is known to be positively associated with aggressive endothelial repair (atherosclerosis) [5], factors that influence the endothelium, which is associated with cardiovascular disease, should be considered as confounding factors in the present analysis. Circulating CD34-positive cell levels determine the association between TG and HDLc on hypertension [19, 20]. In addition, circulating CD34-positive cell levels also act as a determinant factor in the association between chronic kidney disease and atherosclerosis [13]. BMI status has been shown to influence the association between HbA1c and atherosclerosis [21]. Factors such as alcohol consumption and smoking status influence vascular remodeling. Alcohol consumption influences γ-GTP and smoking status influences WBC [22]; therefore, we included γ-GTP and WBC as confounding factors rather than considering these factors directly, as in a previous study [6, 13, 23].

All statistical analyses were performed using the SAS system for Windows (version 9.4; SAS Inc., Cary NC). All *p*-values for statistical tests were two-tailed, and values of < 0.05 were regarded as statistically significant.

## Results

### Characteristics of the study population

Among the study population, 128 participants showed low circulating CD34-positive cells (< 0.96 cells/μL) and 131 were diagnosed as having hypertension. Characteristics of the study population in relation to circulating CD34-positive cell levels are shown in Table 1. Compared to the participants with low circulating CD34-positive cell level, participants with high circulating CD34-positive cells showed a significantly higher rate of taking antihypertensive medication use and higher WBC counts. We also evaluated the correlation between CD34-positive cells and WBC and found no significant correlations; the simple correlation coefficient (r) and *p*-value were *r* = 0.09, *p* = 0.319 for participants with low circulating CD34-positive cell level and *r* = −0.12, *p* = 0.166 for participants with high circulating CD34-positive cell level.

**Table 1:** Characteristics of the study population by CD34-positive cell levels

	CD34-positive cells		*p*
	Low (< 0.96 cells/μL)	High (≥ 0.96 cells/μL)	
Circulating CD34-positive cell, cells/μL	0.63 ± 0.20	2.10 ± 2.06	
No. of participants	128	129	
Age, years	65.6 ± 2.6	65.2 ± 2.5	0.223
Body mass index (BMI), kg/m^2^	23.3 ± 3.0	24.0 ± 2.9	0.093
Daily drinker, %	50.0	48.1	0.757
Current smoker, %	25.8	22.5	0.538
Systolic blood pressure (SBP), mmHg	136 ± 17	137 ± 18	0.549
Diastolic blood pressure (DBP), mmHg	85 ± 12	85 ± 12	0.652
Anti-hypertensive medication (medication), %	39.1	53.5	0.020
Serum HDL-cholesterol (HDLc), mg/dL	57 ± 14	57 ± 14	0.922
Serum triglycerides (TG), mg/dL	101 ± 57	120 ± 93	0.052
Hemoglobin A1c (HbA1c), %	5.6 ± 0.7	5.7 ± 0.6	0.297
Serum γ-glutamyltranspeptidase (γ-GTP), U/L	46 ± 43	45 ± 36	0.922
White blood cells (WBC), cells/μL	5299 ± 1459	6169 ± 1355	< 0.001
Glomerular filtration rate (GFR), mL/min/1.73 m^2^	74.9 ± 14.4	71.9 ± 13.0	0.085
Handgrip strength, kg	38.2 ± 6.4	39.2 ± 5.6	0.177

Values: mean ± standard deviation

### Associations between handgrip strength and hypertension in relation to circulating CD34-positive cell levels

Goodness of fit for the present study population stratified by the status of circulating CD34-positive cell levels was validated. A positive association between handgrip strength and hypertension was found in participants with high circulating CD34-positive cell level, but not in those with low circulating CD34-positive cell level (Table 2). Investigations of the effect of interaction between circulating CD34-positive cell levels and handgrip strength (SD values) on hypertension revealed significant interactions between these two, with *p*-values for the effect of this interaction on hypertension of *p* = 0.039 for the age-adjusted model and *p* = 0.012 for the multivariable model

**Table 2:** Odds ratios (ORs) and 95% confidence intervals (CIs) for hypertension in relation to handgrip strength stratified by circulating CD34-positive cell count

	Handgrip strength levels			*p* for trend	*p* (goodness of fit test)	1 SD increment of handgrip strength (kg)
	T1 (low)	T2	T3 (high)			
**High CD34-positive cell count (≥ 0.96 cells/μL)**						
No. of participants	37	49	43			
No. of cases (%)	14 (37.8)	26 (53.1)	27 (62.8)			
Age-adjusted ORs	1.00	1.87 (0.78, 4.46)	2.81 (1.13, 7.01)	0.027	0.659	1.78 (1.17, 2.71)
Multivariable ORs	1.00	2.20 (0.84, 5.73)	2.92 (1.08, 7.87)	0.037	0.950	1.85 (1.19, 2.88)
**Low CD34-positive cell count (< 0.96 cells/μL)**						
No. of participants	49	39	40			
No. of cases (%)	27 (55.1)	15 (38.5)	22 (55.0)			
Age-adjusted ORs	1.00	0.49 (0.20, 1.19)	0.95 (0.39, 2.31)	0.878	0.864	1.00 (0.71, 1.41)
Multivariable ORs	1.00	0.38 (0.14, 1.03)	0.70 (0.24, 1.98)	0.476	0.858	0.91 (0.61, 1.37)
		Interaction*		*P*		
Age-adjusted model		1.74 (1.03, 2.96)		0.039		
Multivariable model		2.05 (1.17, 3.59)		0.012		

Hypertension is defined as systolic blood pressure ≥ 140 mmHg and/or diastolic blood pressure ≥ 90 mmHg. Multivariable ORs: adjusted further for age and BMI, anti-hypertensive medication, HDLc, TG, γ-GTP, WBC, HbA1c, and GFR. *BMI* body mass index, *HDLc* HDL-cholesterol, *TG* triglycerides, *HbA1c* hemoglobin A1c, *γ-GTP* γ-glutamyltranspeptidase, *WBC *white blood cell, *GFR* glomerular filtration rate. Handgrip strength tertiles are as follows: < 37.0 kg for T1, 37.0–41.9 kg for T2, and ≥ 42.0 kg for T3.*The effect of interaction between circulating CD34-positive cell levels and handgrip strength (1 SD increment: 6.0kg) on hypertension

### Associations between handgrip strength and systolic or diastolic hypertension in relation to circulating CD34-positive cell levels

To investigate the association between systolic and diastolic hypertension and handgrip strength, goodness of fit for the present study population stratified by the status of circulating CD34-positive cell levels was validated. Handgrip strength showed essentially the same associations as systolic hypertension and diastolic hypertension with circulating CD34-positive cell levels (Tables 3 and 4).

**Table 3:** Odds ratios (ORs) and 95% confidence intervals (CIs) for systolic hypertension in relation to handgrip strength stratified by circulating CD34-positive cell count

	Handgrip strength levels			*p* for trend	*p* (goodness of fit test)	1 SD increment of handgrip strength (kg)
	T1 (low)	T2	T3 (high)			
**High CD34-positive cell count (≥ 0.96 cells/μL)**						
No. of participants	37	49	43			
No. of cases (%)	11 (29.7)	22 (44.9)	22 (51.2)			
Age-adjusted ORs	1.00	1.97 (0.80, 4.88)	2.62 (1.03, 6.69)	0.046	0.882	1.81 (1.18, 2.77)
Multivariable ORs	1.00	2.43 (0.88, 6.68)	2.74 (0.97, 7.73)	0.063	0.475	1.88 (1.19, 2.99)
**Low CD34-positive cell count (< 0.96 cells/μL**)						
No. of participants	49	39	40			
No. of cases (%)	24 (49.0)	13 (33.3)	14 (35.0)			
Age-adjusted ORs	1.00	0.48 (0.20, 1.19)	0.51 (0.20, 1.26)	0.135	0.250	0.88 (0.62, 1.26)
Multivariable ORs	1.00	0.43 (0.16, 1.19)	0.41 (0.14, 1.21)	0.098	0.691	0.87 (0.58, 1.33)
		Interaction*		*p*		
Age-adjusted model		1.90 (1.11, 3.25)		0.020		
Multivariable model		2.05 (1.25, 3.89)		0.007		

Systolic hypertension is defined as systolic blood pressure ≥ 140 mmHg. Multivariable ORs: adjusted further for age and BMI, anti-hypertensive medication, HDLc, TG, γ-GTP, WBC, HbA1c, and GFR. *BMI* body mass index, *HDLc* HDL-cholesterol, *TG* triglycerides, *HbA1c* hemoglobin A1c, *γ-GTP* γ-glutamyltranspeptidase, *WBC* white blood cell, *GFR* glomerular filtration rate. Handgrip strength tertiles are as follows: < 37.0 kg for T1, 37.0–41.9 kg for T2, and ≥ 42.0 kg for T3. *The effect of interaction between circulating CD34-positive cell levels and handgrip strength (1 SD increment: 6.0kg) on systolic hypertension

**Table 4:** Odds ratios (ORs) and 95% confidence intervals (CIs) for diastolic hypertension in relation to handgrip strength stratified by circulating CD34-positive cell count

	Handgrip strength levels			*p* for trend	*p* (goodness of fit test)	1 SD increment of handgrip strength (kg)
	T1 (low)	T2	T3 (high)			
**High CD34-positive cell count (≥ 0.96 cells/μL)**						
No. of participants	37	49	43			
No. of cases (%)	10 (27.0)	19 (38.8)	21 (48.8)			
Age-adjusted ORs	1.00	1.69 (0.67, 4.26)	2.48 (0.96, 6.39)	0.060	0.934	1.71 (1.11, 2.64)
Multivariable ORs	1.00	1.61 (0.59, 4.36)	2.58 (0.93, 7.12)	0.066	0.416	1.77 (1.11, 2.82)
**Low CD34-positive cell count (< 0.96 cells/μL)**						
No. of participants	49	39	40			
No. of cases (%)	15 (30.6)	9 (23.1)	18 (45.0)			
Age-adjusted ORs	1.00	0.62 (0.23, 1.68)	1.65 (0.66, 4.15)	0.280	0.854	1.07 (0.74, 1.55)
Multivariable ORs	1.00	0.52 (0.17, 1.60)	1.17 (0.39, 3.54)	0.745	0.891	0.99 (0.64, 1.55)
		Interaction*		*p*		
Age-adjusted model		1.58 (0.91, 2.76)		0.105		
Multivariable model		1.82 (1.03, 3.24)		0.041		

Diastolic hypertension is defined as diastolic blood pressure ≥ 90 mmHg. Multivariable ORs: adjusted further for age and BMI, anti-hypertensive medication, HDLc, TG, γ-GTP, WBC, HbA1c, and GFR. *BMI* body mass index, *HDLc* HDL-cholesterol, *TG* triglycerides, *HbA1c* hemoglobin A1c, *γ-GTP* γ-glutamyltranspeptidase, *WBC* white blood cell, *GFR* glomerular filtration rate. Handgrip strength tertiles are as follows: < 37.0 kg for T1, 37.0–41.9 kg for T2, and ≥ 42.0 kg for T3. *The effect of interaction between circulating CD34-positive cell levels and handgrip strength (1 SD increment: 6.0kg) on diastolic hypertension

Investigations of the effect of interaction between circulating CD34-positive cell levels and handgrip strength (SD values) on systolic hypertension revealed significant interactions between these two, with *p*-values for the effect of this interaction on systolic hypertension of *p* = 0.020 for the age-adjusted model and *p* = 0.007 for the multivariable model (Table 3).

For diastolic hypertension, no significant *p*-value for the interaction between the two was observed (*p* = 0.105); after adjustment for known cardiovascular risk factors, the value became significant (*p* = 0.041) (Table 4).

### Sensitivity analysis

For sensitivity analysis, we repeated the main results by using the drinking status (none, often, daily) and smoking status (never, former, current) as confounding factors instead of using γ-GTP and WBC, and found similar associations. Among participants with high CD34-positive cells, the fully adjusted ORs of hypertension, systolic hypertension, and diastolic hypertension for the 1 SD increment of handgrip strength were 1.87 (1.20, 2.93), 1.97 (1.23, 3.15), and 1.78 (1.13, 2.82). Additionally, among participants with low CD34-positive cells, the corresponding values were 0.90 (0.60, 1.35), 0.86 (0.57, 1.30), and 0.98 (0.64, 1.50), respectively.

## Discussion

The major finding of the present study in older men is that handgrip strength is positively associated with hypertension in participants with high CD34-positive cells, but not in participants with low CD34-positive cells.

Hypertension is a physical stress that injures the endothelium leading to the exposure of sub-endothelial components to the peripheral blood, resulting in platelet activation [24, 25]. These activated platelets elevate circulating CD34-positive cells by stimulating the mobilization of CD34-positive cells from the bone marrow [11, 26–28]. However, under aggressive endothelial repair, many of these cells differentiate into mature cells (CD34-negative cells), which reduce the number of circulating CD34-positive cells. The details of this potential mechanism have been described in Shimizu Y & Maeda T, 2021 [15]. Therefore, physical stress associated with hypertension elevates circulating CD34-positive cells and induces aggressive endothelial repair, which then leads to a reduction in circulating CD34-positive cells. Since antihypertensive medication can reduce physical stress, the use of this medication to treat hypertension should lower the activity of CD34-positive cells in comparison to uncontrolled hypertension or poorly controlled hypertension. In the present study, we focus on the role of CD34-positive cells in endothelial repair. Therefore, we defined hypertension only by the current blood pressure level, similar to in our previous studies [5, 29, 30]. Furthermore, no significant differences were observed in blood pressure levels (both systolic and diastolic), and participants with high CD34-positive cell level showed a higher prevalence of antihypertensive medication use than those with low CD34-positive cell level. This is partly consistent with a previous study that reported that angiotensin II receptor antagonism increases the number of CD34-positive cells [31]. Participants with controlled hypertension might have higher activity of CD34-positive cells than those who do not have hypertension but have a lower rate of CD34-positive cell reduction than those with uncontrolled hypertension. Since 2013, we measured CD34-positive cells in men aged 60–69 years who lived in Goto City and Saza town and found that the rate of uncontrolled hypertension in Goto City is higher than in Saza town; 27.7% and 15.0%, respectively (*p* < 0.001). Unlike Goto City, which is a remote island, Saza Town is located on the main island of Kyusyu. Therefore, the differences in the rate of uncontrolled hypertension may be due to regional variability in muscle strength levels among older participants. Therefore, further investigation of these findings is necessary.

Resistance training in hypoxia and blood flow restriction may augment muscle size and strength development [32, 33]. Hypoxia is known to induce oxidative stress [34], which may function as a key factor in the pathogenesis of hypertension [35]. Hypoxia then induces hypertension [36, 37]. Since aging is a process that has been reported to be associated with oxidative stress [38], the beneficial influence of maintaining muscle strength by using muscles in daily life might be stronger for hypertensive older participants than non-hypertensive participants. Therefore, previous studies revealed a positive association between handgrip strength and blood pressure among older participants [1, 2]. However, in the present study, a significant positive association between handgrip strength and hypertension was observed in participants with high CD34-positive cells but not in participants with low CD34-positive cells.

Previously, muscle and endothelial cell-derived vascular growth factors were shown to regulate crosstalk between blood vessels and muscle cells during regeneration from the ischemic environment [39]. Hypoxic conditions can stimulate angiogenesis and satellite cell activity in mouse skeletal muscle [40]. The CD34-molecule is expressed not only in hematopoietic stem cells but also in non-hematopoietic cells such as muscle satellite cells and vascular endothelial cells [41]. Since hematopoietic stem cells derived from bone marrow play a major role in vascular homeostasis [9–11] and as hematopoietic bone marrow activity declines with age [42, 43], participants with high CD34-positive cells might have a higher capacity for stimulating angiogenesis and activating muscle satellite cells, while showing a lower influence of age-related decline on bone marrow activity than those with low CD34-positive cells.

**Fig. 1:**
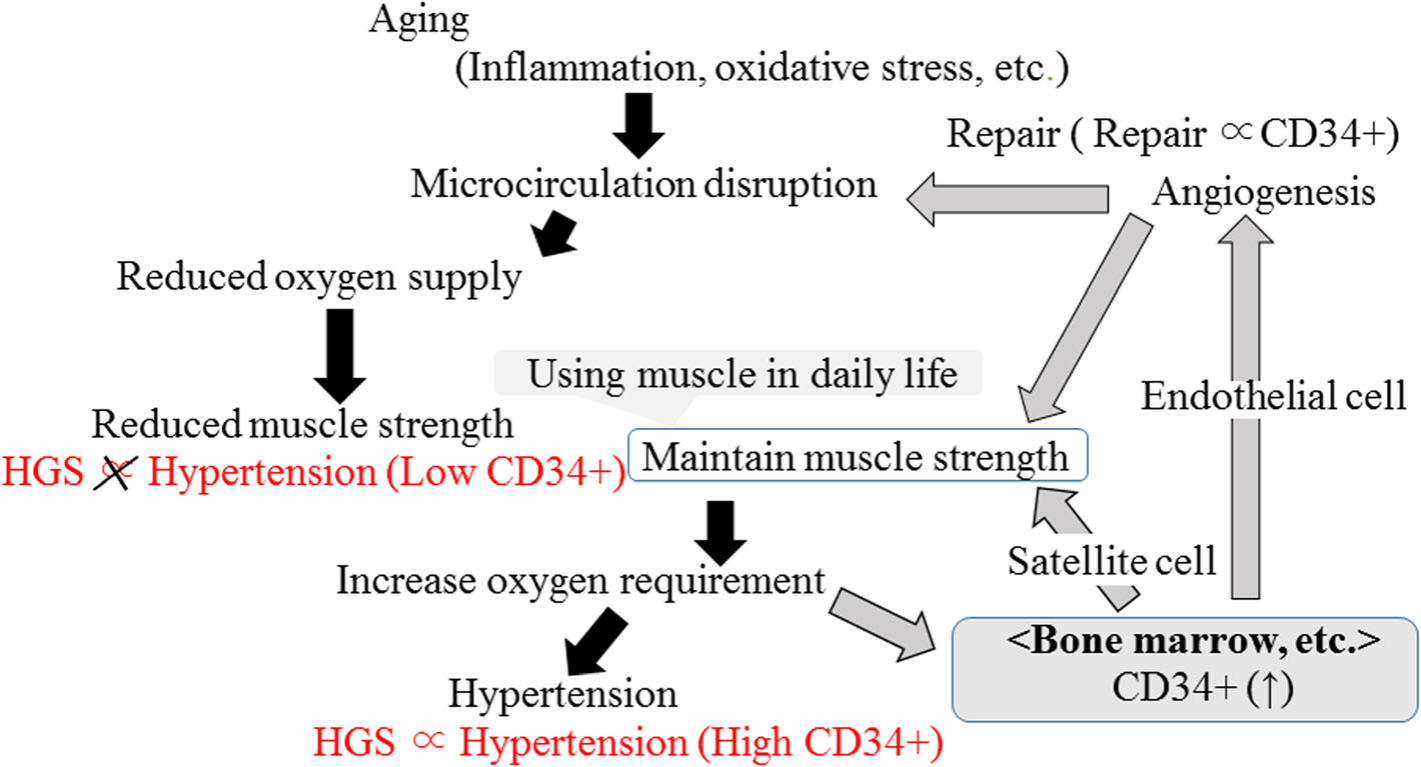
Possible mechanism underlying the association between handgrip strength and hypertension. Associations shown in red were observed in the present study. *HGS* handgrip strength, *CD34+* CD34-positive cell

A summary of the possible mechanisms underlying the association between handgrip strength and hypertension by CD34-positive cell levels is shown in Fig. 1. Age-related changes in microcirculation (disruption) are associated with reduced maximal oxygen supply and exercise capacity [44]. Even using muscles in daily life, which results in maintenance of muscle strength, might increase the requirement for oxygen. To compensate for this increased requirement, hypertension could occur. In addition, comparatively oxygen-deficient conditions stimulate CD34-positive cell production [45], which also could have beneficial effects on maintaining muscle strength.

The clinical implication of the present study is that while hypertension is a well-known cardiovascular risk factor [4], hypertension might have a beneficial association with maintaining muscle strength among older individuals. However, low handgrip strength is a risk factor for cardiovascular diseases [3]. These associations seem paradoxical. CD34-positive cells are also known to contribute to angiogenesis [9], which plays an important role in decreasing blood pressure. Therefore, participants with high CD34-positive cells might possess a higher activity for microcirculation maintenance, which results in a lower prevalence of hypertension [30]. In other words, although further investigation is necessary, hypertensive participants with high CD34-positive cells may reduce their blood pressure in the near future. Since hematopoietic bone marrow activity declines with age [42, 43], the present findings indicate that hematopoietic activity should be taken into consideration when estimating the risk of hypertension among older participants.

Potential limitations of the present study warrant consideration. First, while oxidative stress and requirement of oxygen might play an important role in the present associations, no data concerning these factors were available. Further investigations using data such as those for reactive oxygen species, superoxide dismutase, catalase, and hypoxia-inducing factors are necessary. Even though γ-GTP and WBC were associated with alcohol consumption and smoking status [22], these factors could also be influenced by obesity and other chronic inflammation. However, sensitivity analysis that reruns the main analysis by using drinking status and smoking status as confounding factors instead of using γ-GTP and WBC showed essentially the same associations. Because this was a cross-sectional study, causal relationships could not be established.

## Conclusion

In conclusion, our study revealed that, in addition to well-established cardiovascular risk factors, handgrip strength is positively associated with hypertension in older men with high CD34-positive cells but not with low CD34-positive cells. These results may help to clarify the background mechanism underlying age-related physical changes.

## Data Availability

The datasets generated during and/or analyzed during the current study are not publicly available due to ethical consideration but are available from the corresponding author on reasonable request.

## Abbreviations

SD: Standard deviation
BMI: Body mass index
WBC: White blood cell
TG: Triglycerides
HDLc: HDL-cholesterol
HbA1c: Hemoglobin A1c
γ-GTP: γ-Glutamyltranspeptidase
GFR: Glomerular filtration rate
ISHAGE: International Society of Hematotherapy and Graft Engineering
ANOVA: Analysis of variance
ORs: Odds ratios
CIs: Confidence intervals
